# Distinct Clinical and Experimental Characteristics in the Patients Younger than 60 Years Old with Myelodysplastic Syndromes

**DOI:** 10.1371/journal.pone.0057392

**Published:** 2013-02-28

**Authors:** Xiao Li, Zhi-jian Xiao, Chun-kang Chang, Feng Xu, Ling-yun Wu, Qi He, Ze-feng Xu, Lu-xi Song, Zheng Zhang, Li-yu Zhou, Ji-ying Su, Xi Zhang, Juan Guo

**Affiliations:** 1 Department of Hematology, The Sixth Hospital Affiliated to Shanghai Jiaotong University, Shanghai, China; 2 Department of Clinical Hematology, Institute of Hematology and Blood Diseases Hospital, Chinese Academe of Medical Sciences and Peking Union Medical College, Tianjin, China; University of Medicine and Dentistry of New Jersey, United States of America

## Abstract

Myelodysplastic syndromes (MDS) mainly occur in elderly individuals in Western countries. However, MDS is commonly found in young individuals (<60 years) in Asia. The reason for the high incidence in younger individuals is still unclear, and the differences in disease features between young and elderly patients with MDS have been not well recognized. To explore these issues, in this study, we analyzed the clinical and experimental characteristics of MDS in the patients younger and older than 60 years old and characterized the potential age-associated differences. The results showed that over half of the patients with MDS (61.9%) were younger than 60 years old upon the first diagnosis. The younger patients were more likely to be female, who have lower risk and less advanced MDS. The occurrence of trisomy 8 and bone marrow failure were more frequent in the younger patients than the older ones. The marrow CD34+ cells in the younger patients showed lower proliferation and higher apoptosis in comparison with that in the older ones. Obvious amplification of T cells and low CFU formation could be found in the younger patients. CFU formation was significantly increased in the younger patients after the removal of activated T cells. In addition, the younger patients had a lower frequency of p15^INK4B^ methylation, longer survival expectancy and less AML transformation. In summary, the younger patients with MDS in China may show more benign disease features than the older ones. Enhanced immunological response may be involved in the pathogenesis of MDS in the patients younger than 60 years.

## Introduction

In Western countries, myelodysplastic syndromes (MDS) occur more frequently in older individuals [1]–[4]. The MDS incidence increases sharply with age, with an incidence of 15.0–22.8 per 100,000 per year for patients in their 70s [4]. A large-sample analysis from Germany (including 2728 cases) showed that the median age at diagnosis for MDS patients was 71 years old [5]. In Asia (e.g., Japan, Thailand and South Korea), MDS has been reported to occur more frequently in younger individuals, with the median age of 50–60 years at diagnosis [6]–[8]. Chen et al. [9] and Li et al. [10] released two independent analysis regarding 508 and 351 cases of de novo MDS in the Chinese population respectively, with the median age of 49 and 45 years, respectively. There are few studies on the association between age and disease characteristics in MDS. Kuendgen et al [5] first performed a comparative analysis between MDS patients younger and older than 50 years to screen the candidates for allo-PBSCT. The results showed that the patients younger than 50 years old included more females, and the younger patients (<50 years old) had significantly longer survival period than the older ones in low-risk group. However, no more difference in the clinical and experimental characteristics between the two groups was described.

In this study, we performed a retrospective analysis to investigate whether a difference in disease features do exist between young and elderly patients with MDS. In addition, through this study, we sought to investigate whether different disease characteristics in younger patients imply distinct pathogenesis. The choice of the cut-off age between young and elderly patients is an important issue. WHO and UN recommended that the cut-off age of the elderly could be 60 years old [11], [12]. In addition, 60 years old is also considered as candidate cut-off for immunosuppressive therapy or HSCT [13]. Herein, we referred to 60 years as the cut-off between younger and older patients. More parameters, such as HLA-DR15, feature analysis of the CD34+ cells, T cell subsets and polarization, CFU growth after the removal of activated T cells, and p15^INK4b^ methylation, were also integrated into this study. Through this study, we could deepen the insight into the age-associated pathogenesis of MDS.

## Materials and Methods

### Patients

502 patients with MDS between January, 2006 and April, 2011 were included in this study. The MDS diagnoses were made in accordance with the minimum diagnostic criteria [14]. More than half of patients in this study only accepted best supportive treatment. Some other patients accepted immunosuppressive therapy (n = 71), decitabine (n = 62) or chemotherapy (n = 95). Just a few patients (n = 5) accepted PBSCT. Non-MDS diseases were excluded based on clinical characteristics, morphological changes, special biochemical indicators and the response to treatment. All clinical and laboratory data involved in this study from MDS patients were acquired at the first visit except for overall survival (OS) and leukemia-free survival (LFS). The OS or LFS data for the patients who accepted PBSCT was censored at the time of treatment.

### Ethics Statement

All subjects provided written informed consent. The written informed consent was obtained from patient himself (if minors/children participants, written informed consent was obtained from their guardians). The study was approved by the Ethics Committee of the Sixth Hospital affiliated with Shanghai Jiao Tong University, and Chinese Academe of Medical Sciences, Tianjin, China. All patient-relevant research strictly abided by the Declaration of Helsinki.

### MDS Classification

MDS was classified according to the WHO criteria (2008) [15] except some particular subsets such as chronic myelomonocytic leukemia (CMML) and refractory anemia with excessive blasts in transformation (RAEB-t). CMML and RAEB-t were defined according to the FAB classification [16]. The international prognostic scoring system (IPSS) was used as previously described [17].

### Peripheral Blood and Bone Marrow Examination

Complete blood cell counts and bone marrow analysis were performed for each patient. At least 500 nucleated cells from bone marrow aspirates were counted and analyzed. The percentage of marrow blasts in all nucleated cells was calculated. In calculating, nucleated erythroid cells should be excluded when their percentage exceeded 50% in all nucleated cells [16]. Blasts and ringed sideroblasts were strictly defined according to the consensus proposals suggested by the International Working Group on Morphology of Myelodysplastic Syndrome (IWGM-MDS) [18]. Plastic-embedded BM biopsies were prepared to evaluate the marrow cellularity and to rule out other diagnoses. A level of hematopoietic tissues below 30% in tissue sections was defined as marrow hypocellularity.

### Chromosome Analysis

A routine G-Banding analysis was conducted for all patients [19]. When fewer than 10 metaphase cells were present, fluorescence in situ hybridization (FISH) [20] was performed using four probes LSI(5q31)(D5S721), LSI(20q12)(D20S108), CEP8D8Z2 and CEP7D7Z1) to assess the common abnormalities such as 5q−, 20q−, +8, and −7.

### Detection of HLA-DR15

In brief, as previously described [21], 2–3 ml of peripheral blood or bone marrow was collected. The DNA was extracted using the QIAamp DNA Blood Mini Kit (Qiagen, Germany). The PCR mixture was performed as follows: 0.3 µl of the primers, 2 µl of 10 x Taq buffer, 1 µl of dNTPs (10 mM), 2 µl of MgCl_2_ (25 mM), 1 unit of Taq DNA polymerase (MBI Fermentas, USA), and 0.05 µg of DNA. The final volume was then adjusted to 20 ul with deionized water. PCR amplification was performed using a PTC-200 thermocycler (MJ Research, USA). The PCR products were then analyzed with 2% agarose gel electrophoresis.

### Immunophenotyping and Apoptosis Analysis of BM CD34+ Cells

The following fluorescent-labeled antibodies were used: CD45, CD34, CD19, CD38, CD117 and CD7. The expression of CD19, CD38, CD117 and CD7 in CD34+ cells are considered to be related to differentiation and proliferation of HSCs [19]–[22]. The percentage of CD34+CD19+ cells (refer to B-cell progenitors) reflects the differentiation from HSCs to B cells [22]. CD34+ cells with low CD38 expression represents early or low-differentiation HSCs [23]. CD117 and CD7 expression reflects higher proliferation degree of HSCs [24], [25]. The following sets of detections were used: CD45/CD34/CD19/CD38, CD45/CD34/CD7/CD117 and IgG1-PerCP/APC/FITC/PE (isotype control). All antibodies were purchased from BD Biosciences Heparin anticoagulant marrow solutions were treated with NH_4_Cl (hemolytic reagent) and total bone marrow nucleated cells were subsequently isolated. All samples underwent FCM within 6 hours. A flow cytometer (FACS Calibur, Becton Dickinson) equipped with CellQuest software was used for logarithmic (Log) sampling, in which at least 10^5^ total cells were acquired and analyzed. CD45/SSC gating was configured to delimit the population of leucocytes; CD34+ blasts with immunophenotypes of CD45^int^CD34^int/high^SSC^low^ were screened to delimit, followed by the analysis of the expression of surface antigens (CD19, CD38, CD117 and CD7). For apoptosis detection, BM cells were stained with anti-CD34- APC at room temperature for 20 minutes and then were labeled with 5 µL of Annexin V-FITC (BD Biosciences) in combination with 1 µL of PI for 15 minutes. After incubation, the cells were washed once and were analyzed within 2 hours.

### Subset and Polarization of BM Lymphocytes

In brief, as previously described [26], bone marrow mononuclear cells (BMMCs) were incubated with phorbol 12-myristate 13-acetate (PMA) and ionomycin (Sigma, USA) at 37°C for 4 h. The cells were then stained with anti-CD3-PerCP and anti-CD8-APC (BD, USA) for 15 mins. After treatment with IntraPreP permeabilization reagent B (BD, USA), the cells were stained with anti-human IFN-γ-FITC and IL-4-PE (BD, USA) for 15 mins. All antibodies were purchased from BD Biosciences. Flow cytometry was performed using FACS Calibur, and the results were analyzed with CellQuest software. The T-helper (Th) and T-cytotoxic (Tc) subsets were classified as Th1 (CD8− INF-γ+), Th2 (CD8− IL-4+), Tc1 (CD8+ INF-γ+) and Tc2 (CD8+ IL-4+).

### In vitro Colony Formation Assay

The CD8+CD57+ and CD4+CCR5+ cells were defined as activated CD8+ and CD4+ T cells, respectively [27], [28]. The CD8+CD57+ and CD4+CCR5+ cells were sorted by magnetic cell separation from BMMCs. 1.2×10^6^ of BMMCs and 1.2×10^6^ of BMMCs without CD8+CD57+ cells or CD4+CCR5+ cells were resuspended in 400 µL of a resuspension solution (R&D Systems, USA). In all cases, semi-solid culture were performed according to the manufacturer’s instructions (R&D Systems) for growing colony-forming units (CFUs). The culture was incubated at 37°C in a fully humidified, 5% CO2-containing atmosphere, and the colony formation was analyzed in the presence and absence of CD8+CD57+T or CD4+/CCR5+ cells after 14 days. Colonies were scored as aggregates with more than 40 cells.

### Methylation of P15^INK4B^

A 1 µg DNA sample in a 100-µl volume was treated with bisulfite using the EZ DNA Methylation™ Kit (Zymo Research, CA) according to the manufacturer’s instructions. The modified DNA was subjected to two separate rounds of PCR. As previously described [29], MS-PCR primers were designed to amplify the methylated (M) or unmethylated (U) alleles. The 10-µl PCR mixture contained 80 µg of bisulfite-treated DNA, 1 unit of AccuPrime Taq DNA Polymerase (Invitrogen, UK), and 2.5 mmol/L of each primer. The PCR products were electrophoresed on 10% polyacrylamide gels and visualized with ethidium bromide staining and ultraviolet transillumination.

### Patient Survival

The comparison of overall survival and leukemia-free survival between the younger patients and the older ones were analyzed according to the IPSS prognosis, FAB/WHO classification and cytogenetic risk groups.

### Statistical Analysis

All data were analyzed with the tool of SPSS 11.0 statistical software package. A cohort comparison was performed by use of Pearson’s χ2 or Fisher’s exact test for categorical data, and the paired measurement data were analyzed using Student’s t-test. Kaplan-Meier curves were used for analysis of overall survival (OS) and leukemia-free survival. The comparison of survival rate and AML transformation rate between the younger MDS patients (<60 years old) and the older ones (≥60 years old) was analyzed using the log rank test. Difference with a P-value of <0.05 was considered to be statistically significant.

## Results

### Patient Characteristics

**Figure 1:**
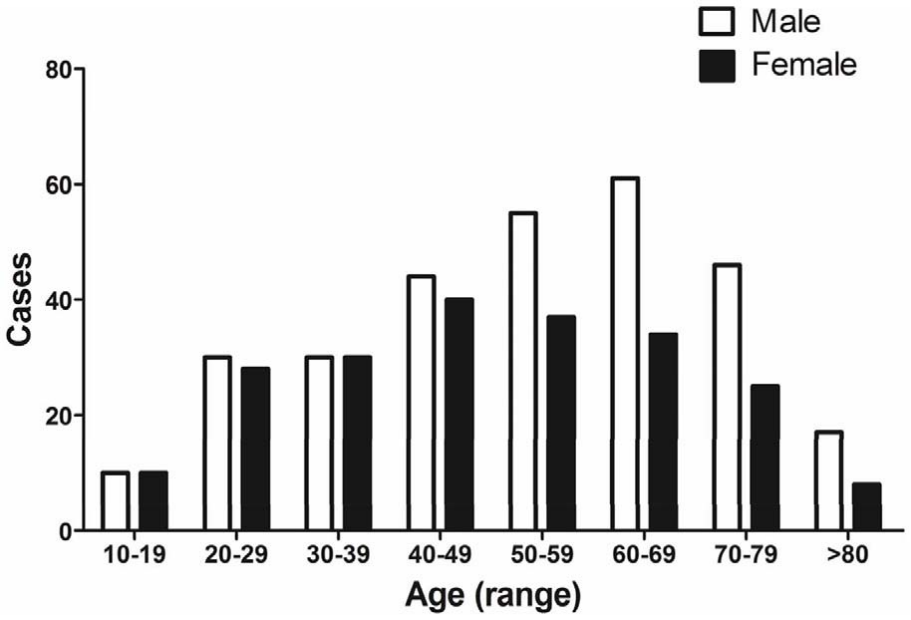
Distribution of age and gender in MDS patients. Male-to-female ratio in MDS patients gradually increases from the young to the old. Similar incidence of MDS could be observed in the younger male and female patients before age 60. However, the older male patients have higher incidence of MDS compared with the older female patients.

As shown in Table 1, a total of 502 patients were analyzed. The male-to-female ratio was 1.37∶1, and the median age was 53 years (range, 13–89 years). 311 patients were younger than 60 years old, and the median age was 44 years (range, 13–59 years); 191 patients were older than 60 years, and the median age was 71 years (range, 60–89 years). The younger group accounted for 61.9% of all MDS patients and had a male-to-female ratio of 1.15∶1; the older group accounted for 38.1% of all patients and had a male-to-female ratio of 1.85∶1 (*P* = 0.011, compared with the younger group). Apparently, over half of MDS patients were younger than 60 years, and male-to-female ratio in MDS patients gradually increases from the young to the old (Figure 1).

**Table 1:** Clinical characteristics of both younger and older MDS patients.

Variable	Total(n = 502)	<60(n = 311)	≥60(n = 191)	P-Value
**Sex ratio (male: female)**	1.37∶1 (290/212)	1.15∶1 (166/145)	1.85∶1 (124/67)	0.011
**Age, years (range)**	53 (13–89)	44 (13–59)	71 (60–89)	<0.001
**WHO/FAB classification**				
*Less advanced MDS*	63.5 (319/502)	70.4 (219/311)	52.4 (100/191)	<0.001
RN+RT	4.4 (22/502)	5.5 (17/311)	2.6 (5/191)	0.148
RA	5.8 (29/502)	5.5 (17/311)	6.3 (12/191)	0.640
RARS	2.8 (14/502)	1.3 (4/311)	5.2 (10/191)	0.009
RCMD	40.2 (232/502)	55.6 (173/311)	30.9 (59/191)	<0.001
RCMD-RS	3.6 (18/502)	2.6 (8/311)	5.2 (10/191)	0.119
5q-syndromes	0.8 (4/502)	0 (0/311)	2.1 (4/191)	0.005
*Advanced MDS*	36.5 (183/502)	29.6 (92/311)	47.6 (91/191)	<0.001
RAEB I	14.7 (74/502)	12.2 (38/311)	18.8 (36/191)	0.042
RAEB II	9.8 (49/502)	8.4 (26/311)	12.0 (23/191)	0.177
RAEB-T	6.8 (34/502)	7.7 (24/311)	5.2(10/191)	0.283
CMML	5.2 (26/502)	1.3 (4/311)	11.5 (22/191)	<0.001
**IPSS category**				
Lower-risk (≤1.0)	69.7 (350/502)	74.3 (231/311)	62.3 (119/191)	0.005
Higher-risk (≥1.5)	30.3 (152/502)	25.7 (80/311)	37.7 (72/191)	0.005
**Abnormal chromosomes**				
5q−/−5	16.3 (34/209)	9.8 (11/112)	23.7 (23/97)	0.007
20q−/−20	17.7 (37/209)	12.5 (14/112)	23.7 (23/97)	0.034
Trisomy 8	32.1 (67/209)	41.1 (46/112)	21.6 (21/97)	0.010
7q−/−7	15.3 (32/209)	13.4 (15/112)	17.5 (17/97)	0.408
Complex karyotypes	25.4 (53/209)	27.7 (31/112)	22.7 (22/97)	0.362
Total	41.6(209/502)	36.0 (112/311)	50.8 (97/191)	<0.001
**Peripheral blood analysis**				
Hb (g/L)	77 (32–150)	78 (32–150)	76 (40–140)	0.473
Neutrophil (×10^9^)	1.4 (0.2–18.3)	1.4 (0.2–6.1)	1.7 (0.3–18.2)	0.002
PLT (×10^9^)	75 (5–680)	60 (5–410)	101 (6–680)	<0.001
**Lineages of Cytopenias**				
0–1	17.9 (90/502)	14.8 (46/311)	23.0 (44/191)	0.019
2–3	82.1 (412/502)	85.2 (265/311)	77.0 (147/191)	0.019
**Hypocellularity (%)(cases)**	20.7 (104/502)	25.9 (81/311)	12.0(23/191)	0.001
**HLA-DR15 positive (%)(cases)**	27.5(87/316)	27.4 (58/212)	27.9 (29/104)	0.922
**p15 methylation (%) (cases)**	71.2 (272/382)	64.4 (152/236)	82.2 (120/146)	0.005

### FAB/WHO Classification of MDS

FAB and WHO 2008 classification were used to classify MDS. RCUD (RA/RN/RT), RARS, RCMD and 5q− syndrome were defined as less advanced MDS; RAEB-1, RAEB-2, RAEB-T and CMML were defined as advanced MDS. Less advanced MDS accounted for 70.4% and 52.4% of the cases in the younger and the older group, respectively, while advanced MDS accounted for 29.6% and 47.6% of the cases in the younger and the older group, respectively (Table 1). The percentage of the younger patients is significantly higher than that of the older ones in less advanced MDS (*P*<0.001). On the contrary, the percentage of the older patients is significantly higher than that of the younger ones in advanced MDS (*P*<0.001). As far as single MDS subtype was concerned, the percentage of the younger patients is significantly higher than that of the older ones in MDS with RCMD (*P*<0.001), whereas the percentage of the older patients is significantly higher than that of the younger ones in MDS with RARS, 5q− syndromes or CMML (*P* = 0.009; *P* = 0.005; *P*<0.001). There was no obvious difference between the younger and the older patients in other subsets of MDS.

### IPSS Classification

The patients with IPSS scores of ≤1.0 and IPSS scores of ≥1.5 are defined as lower-risk and higher-risk patients, respectively. The percentage of the younger patients is significantly higher than that of the older ones in lower-risk MDS (74.3% VS 62.3%, *P* = 0.005). On the contrary, the percentage of the older patients is obviously higher than that of the younger ones in higher-risk MDS (25.7% VS 37.7%, *P* = 0.005) (Table 1 ).

### Karyotypes

As shown in Table 1, the karyotype analysis showed that 112 of 311 patients (36.0%) in the younger group had clonal chromosome abnormalities, which was significantly lower than the percentage (50.8%, 97 of 191 cases) in the older group (*P*<0.001). Among these common chromosome abnormalities, trisomy 8 was observed more frequently in the younger patients than the older ones (41.1% VS 21.6%, *P = *0.010). Abnormalities of chromosome 5 or 20 was less found in the younger patients compared with the older ones (*P* = 0.007; *P* = 0.034). Incidence of abnormalities of chromosome 7 or complex karyotypes showed no significant difference between the younger and older patients. Trisomy 8 was the most frequent chromosomal abnormality in the younger patients, whereas −5 and 5q− were the most frequent abnormalities in older ones.

### Cytopenias and BM Cellularity

There was no significant difference in hemoglobin level between the younger and older patients. However, the younger MDS patients showed lower WBC and PLT count than the older ones (*P* = 0.002; *P*<0.001) (Table 1). The percentage of patients with bi- or pan-cytopenias was higher in the younger group than the older group (85.2% VS 77.0%, *P* = 0.019). In addition, the younger patients showed more frequent BM hypocellularity (hematopoietic tissue less than 30%) in marrow biopsies than the older ones (25.9% VS 12.0%, *P* = 0.001) (Table 1). In brief, more frequent incidence of BM failure could be observed in the younger patients than the older ones.

### Expression Analysis of HLA-DR15

Among the 316 patients with HLA-DR15 available, 212 younger patients and 104 older ones were examined for the HLA-DR15 allele. 58 cases of 212 (27.4%) younger patients and 29 cases of 104 (27.9%) older ones have positive HLA-DR15 allele. No significant difference was found between the two groups (Table 1).

### p15^INK4B^ Methylation

Among the 382 patients with p15^INK4B^ methylation analysis available, 236 cases of younger patients and 146 cases of older patients were determined for p15^INK4B^ methylation. As shown in Table 1, p15^INK4B^ methylation was documented in 64.4% and 82.2% of the younger and older patients, respectively. The difference was of statistical significance (*P* = 0.005).

### Immunophenotyping and Apoptosis Analysis of BM CD34+ Cells

To investigate the proliferation, differentiation and apoptosis of CD34+ cells between the MDS younger patients and the older ones, some surface antigens that reflect proliferation, differentiation and apoptosis were entailed in this study. As presented in Table 2, 158 younger patients and 107 older ones were determined for differentiation, proliferation, and apoptosis markers. Lower percentage of CD34+ cells is observed in the younger patients than the older ones (0.49% VS 2.41%, *P* = 0.045). The percentage of CD34+CD19+ cells was significantly higher in the younger patients than the older ones (8.79% VS 3.53%, *P*<0.001). The younger patients showed lower expression of CD117 in CD34+ cells than the older ones (85.17% VS 93.78%, *P* = 0.039). The expression of CD38 and CD7 in CD34+ cells showed no significant difference between the younger and the older ones. In addition, apoptosis of CD34+ cells was also analyzed by FCM. The younger patients showed significantly higher apoptosis of CD34+ cells (Annexin V+) than the older ones (21.3% VS 8.6%, *P*<0.001). In brief, besides lower percentage in BM, CD34+ cells in the younger patients have better B-cell differentiation, lower proliferation, and higher apoptotic rate.

**Table 2:** FCM analysis of CD34+ cells and lymphocytes in both groups of MDS patients.

FCM parameters	Patients <60 years old	Patients ≥60 years old	*P*-Value
**CD34+ cells immunophenotypes**	**n = 158**	**n = 107**	
% of CD34+ cells (range)	0.49 (0.03–21.55)	2.41 (0.15–18.64)	0.045
% of CD19/CD34 cells (range)	8.79 (0.30–66.51)	3.53 (0.46–42.34)	<0.001
CD38 RMFI of CD34+ cells (range)	268.48 (29.20–1314.61)	217.26 (29.46–1186.45)	0.407
% of CD117/CD34 (range)	85.17 (18.96–99.67)	93.78 (29.46–99.60)	0.039
% of CD7/CD34 (range)	12.50 (0.54–78.85)	8.64 (0.54–79.30)	0.154
Apoptosis (% ) of CD34+ cells (range)	21.3 (2.7–72.3)	8.6 (1.9–68.5)	<0.001
**Lymphocyte immunophenotypes**	**n = 236**	**n = 146**	
% of CD3+T cells	69 (21–98)	63 (12–93)	0.067
% of CD3+CD4+T cells	30 (3–56)	29 (10–65)	0.409
% of Th1 cells	11.0 (0.2–49.0)	12.0 (0.3–55.0)	0.512
% of Th2 cells	0.5 (0.1–10.0)	0.7 (0.1–13.0)	0.260
% of CD3+CD8+T cells	34 (10–64)	29 (4–62)	0.038
% of Tc1 cells	25.6 (0.2–77)	37.8 (1.3–85.0)	0.001
% of Tc2 cells	0.3 (0.1–7.2)	0.5 (0.1–14.3)	0.179
% of CD16/56 (NK cells)	10 (1–43)	14 (2–58)	0.001
% of CD19 (B cells)	9.0 (0.3–37.8)	7.3 (1.0–32.4)	0.268

### Immunophenotyping and Polarization Analysis of BM Lymphocytes

**Figure 2:**
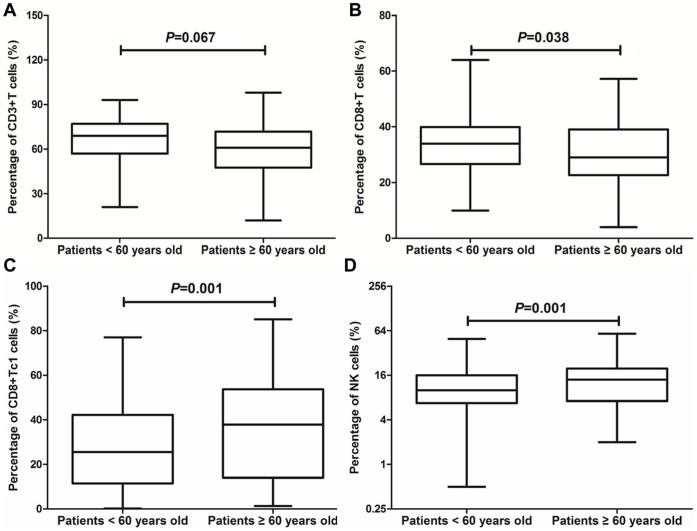
Comparison analysis in BM lymphocytes between the younger and older patients with MDS patients. Compared with the older patients with MDS, the younger ones showed higher percentage of total T cells (CD3+ cells) (**A**) and CD8+ T cells (**B**) (*P* = 0.067; *P* = 0.038). However, the older patients showed higher percentage of CD8+ Tc1 cells (CD8+IFN-γ+ cells) (**C**) and NK cells (CD16+ or 56+ cells) (**D**) than the younger ones (*P* = 0.001; *P* = 0.001).

Among the 382 patients with lymphocyte immunophenotyping analysis available, 236 younger patients and 146 older ones were determined for lymphocyte immunophenotypes (Table 2). The percentage of total T cells (CD3+T cells) was slightly higher in the younger patients than the older ones, but the difference was not statistically significant (69% VS 63%, *P* = 0.067) (Figure 2A). The percentage of cytotoxic T cells (CD8+T cells) was significantly higher in the younger patients than the older ones (34% VS 29%, *P* = 0.038) (Figure 2B). However, the percentage of Tc1 cells and NK cells were higher in the older patients than the younger ones (37.8% VS 25.6%, *P* = 0.001; 14% VS 10%, *P* = 0.001) (Figure 2C and 2D). The percentage of helpful T cells, Th1, Th2, Tc2 and B cells were not significantly different between the younger patients and older patients (Table 2).

### *In vitro* Colony Formation of BM Mononuclear Cells in the Absence or Presence of Activated T Cells

Colony formation of BMMCs was studied in 70 cases with younger patients and 55 cases with older ones (Table 3). In vitro routine culture (activated T cells weren’t removed), colony formation (Mean ± SD) showed no significant difference between the younger and the older ones (29.0±8.5 VS 34.9±9.7, *P* = 0.154). After removal of activated CD4+T cells, the younger patients showed significantly higher colony formation than the older ones (38.1±9.4 VS 25.0±8.6, *P* = 0.001). Similarly, the younger patients showed significantly higher colony formation than the older ones after removal of activated CD8+T cells (48.2±9.5 VS 34.6±9.2, P = 0.001). Although the older patients showed increased ratio of lymphocytes subsets (high percentage of Tc1 and NK cells) which may target towards tumor cells than that in the younger patients, the removal of activated T lymphocytes did not caused increased colony formation of hematopoietic cells in the older patients.

**Table 3:** In vitro colony formation assay of both younger and older MDS patients.

Colony formation	Patients <60 years old (n = 70)	Patients ≥60 years old (n = 55)	*P*-Value
**Routine culture**			
CFU-GM	16.7±7.5	20.6±8.8	0.108
CFU-E	7.1±3.9	6.4±2.3	0.674
CFU-GME	5.2±2.8	7.9±3.8	0.731
Total	29.0±8.5	34.9±9.7	0.154
**Removal of activated CD4+ T cells**			
CFU-GM	25.5±10	14.7±9.4	0.020
CFU-E	6.5±4.6	5.7±3.2	0.588
CFU-GME	6.1±4.0	4.6±2.0	0.159
Total	38.1±9.4	25.0±8.6	0.001
**Removal of activated CD8+ T cells**			
CFU-GM	31.1±12.1	20.1±8.6	0.007
CFU-E	10.3±5.1	7.8±3.6	0.114
CFU-GME	6.9±3.9	6.7±3.6	0.854
Total	48.2±9.5	34.6±9.2	0.001

### Comparison of OS between the Younger Patients and the Older Ones with MDS

**Figure 3:**
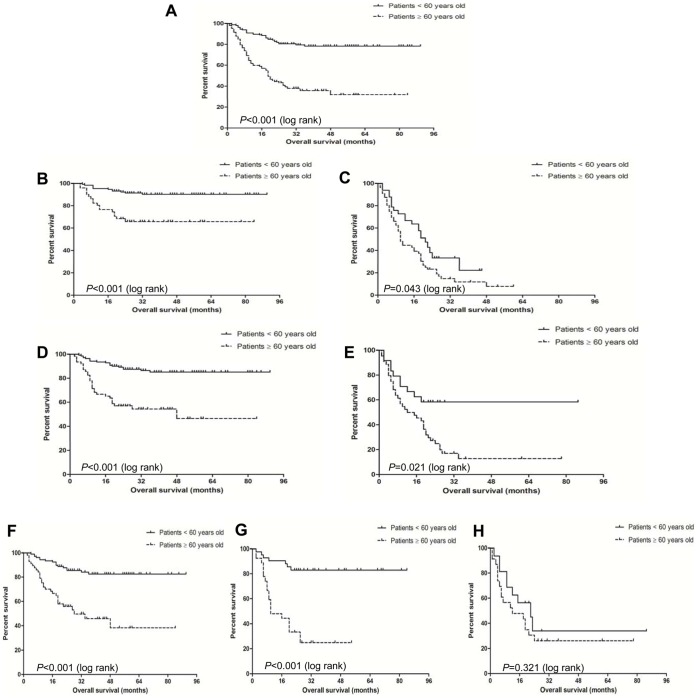
Comparison of survival analysis between the younger patients and the older ones with MDS. The younger patients showed significantly longer overall survival (OS) than the older ones (P<0.001) (**A**). According to WHO classification, the younger patients with less advanced MDS had significantly longer OS than the older ones with less advanced MDS (P<0.001) (**B**); the younger patients with advanced MDS also showed significantly longer OS than the older ones (*P* = 0.043) (**C**). According to IPSS grouping, the younger patients with lower-risk showed significantly longer OS than the older ones with lower-risk (*P*<0.001) (**D**); the younger patients with higher-risk also showed significantly longer OS than the older ones (*P* = 0.021) (**E**). According to karyotype prognosis scoring, the younger patients with good karyotype showed significantly longer OS than the older ones (*P*<0.001) (**F**); Similarly, the younger patients with intermediate-risk karyotype showed significantly longer OS than the older ones (*P*<0.001) (**G**). However, there is no difference in OS between the younger patients and the older ones with poor karyotype (*P* = 0.321) (**H**).

The median OS was not reached in both groups. However, the younger patients showed significantly longer OS than the older ones (P<0.001), and the probability for survival at 48 months was 73.8% and 34.3% respectively in the younger group and the older one (Figure 3A). The life expectancy of the younger and the older patients were compared in accordance with different risk groups such as WHO classification, IPSS and karyotype prognosis grouping. According to WHO classification, the younger patients with less advanced MDS had significantly longer OS (median OS not reached) than the older ones with less advanced MDS (median OS not reached) (P<0.001) (Figure 3B). Similarly, the younger patients with advanced MDS also showed significantly longer OS than the older ones (median OS: 19 VS 16 months) (*P* = 0.043) (Figure 3C). According to IPSS grouping, the younger patients with lower-risk showed significantly longer OS (median OS not reached) than the older ones with lower-risk (median OS is 20 months) (*P*<0.001) (Figure 3D). The younger patients with higher-risk also showed significantly longer OS than the older ones (median OS: 19 VS 12 months) (*P* = 0.021) (Figure 3E). According to karyotype prognosis scoring, the younger patients with low-risk karyotype showed significantly longer OS (median OS not reached) than the older ones with low-risk karyotype (median OS is 28 months) (*P*<0.001) (Figure 3F). The younger patients with intermediate-risk karyotype showed significantly longer OS (median OS not reached) than the older ones (median OS is 10 months) (*P*<0.001) (Figure 3G). However, there is no difference in OS between the younger patients and the older ones with high-risk karyotype (median OS: 20 VS 13 months) (*P* = 0.321) (Figure 3H).

### Comparison of LFS and AML Transformation between the Younger Patients and the Older Ones with MDS

**Figure 4:**
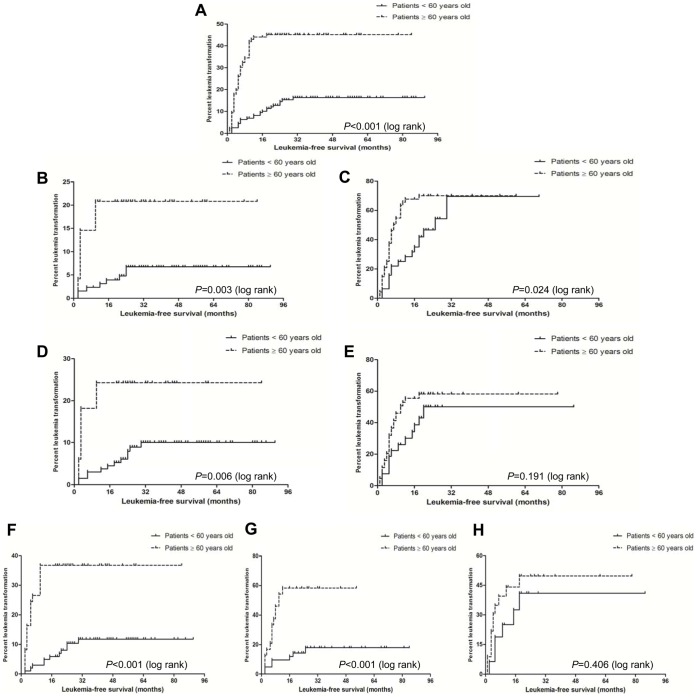
Comparison of LFS between the younger patients and the older ones with MDS. The younger ones had significantly longer LFS than the older ones (*P*<0.001) (**A**). According to WHO classification, whether the younger patients with less advanced (**B**) or advanced MDS (**C**) had significantly longer LFS than the older ones with less advanced or advanced MDS (*P* = 0.003; *P* = 0.024). According to IPSS grouping, the younger patients with lower-risk had significantly longer LFS than the older ones with lower-risk (*P = *0.006) (**D**). However, there is no difference in LFS between the younger and the older patients with higher-risk (*P = *0.191) (**E**). According to karyotype prognosis grouping, whether the younger patients with good (**F**) or intermediate (**G**) karyotype had significantly longer LFS than the older ones with good or intermediate karyotype (*P*<0.001; *P*<0.001). However, there is no difference in LFS between the younger and older ones with poor karyotype (*P = *0.406) (**H**).

In all MDS patients, the younger ones had significantly longer LFS than the older ones (P<0.001), and the AML transformation rate at 24 months was 12.1% and 44.2% respectively in the younger group and the older one (Figure 4A). The LFS in the younger and the older groups was also compared based on WHO classification, IPSS and karyotype prognosis grouping. According to WHO classification, whether the younger patients with less advanced or advanced MDS had significantly longer LFS than the older ones with less advanced or advanced MDS (*P* = 0.003; *P* = 0.024). Whether classifying according to less advanced or advanced MDS, the AML transformation rate at 24 months was significantly lower in the younger group than the older ones (5.2% VS 20.8% and 44.2% VS 69.8%) (Figure 4B and 4C). According to IPSS grouping, the younger patients with lower-risk had significantly longer LFS than the older ones (*P = *0.006), and the AML transformation rate at 24 months was significantly lower in the younger group than the older one (5.4% VS 24.6%) (Figure 4D). However, there is no difference in LFS between the younger and the older patients with higher-risk (*P = *0.191), and the AML transformation rate at 24 months was 49.7% and 58.2% respectively in the younger group and the older one (Figure 4E). According to karyotype prognosis grouping, whether the younger patients with good or intermediate karyotype had significantly longer LFS than the older ones (*P*<0.001; *P*<0.001). Whether classifying according to good or poor karyotype, the AML transformation rate at 24 months was lower in the younger group than the older one (7.7% VS 37.1% and 17.5% VS 58.8%) (Figure 4F and 4G). However, there is no difference in LFS between the younger and older ones with poor karyotype (*P = *0.406), and the AML transformation rate at 24 months was 40.2% and 51.1% respectively in the younger group and the older one (Figure 4H).

## Discussion

The purpose of this study is to explore whether there are some different clinical/experimental characteristics between the younger MDS patients and the older ones and whether these characteristics imply distinct age-associated pathogenesis of MDS. The large-sample data in this study indeed presented distinct clinical and experimental characteristics of the younger MDS patients compared with the older ones. As shown in this study, besides the similar features reported by Kuendgen et al [5], the MDS patients younger than 60 years old had more frequent bone marrow failure, trisomy 8, lower risk and less advanced MDS compared with those older than 60 years old. In addition, the younger patients also showed higher apoptosis of hematopoietic cells, abnormal T-cell activation and lower frequency of p15^INK4B^ methylation. All these distinct findings provided some important clues to explore the pathogenesis of the younger MDS patients.

**Figure 5:**
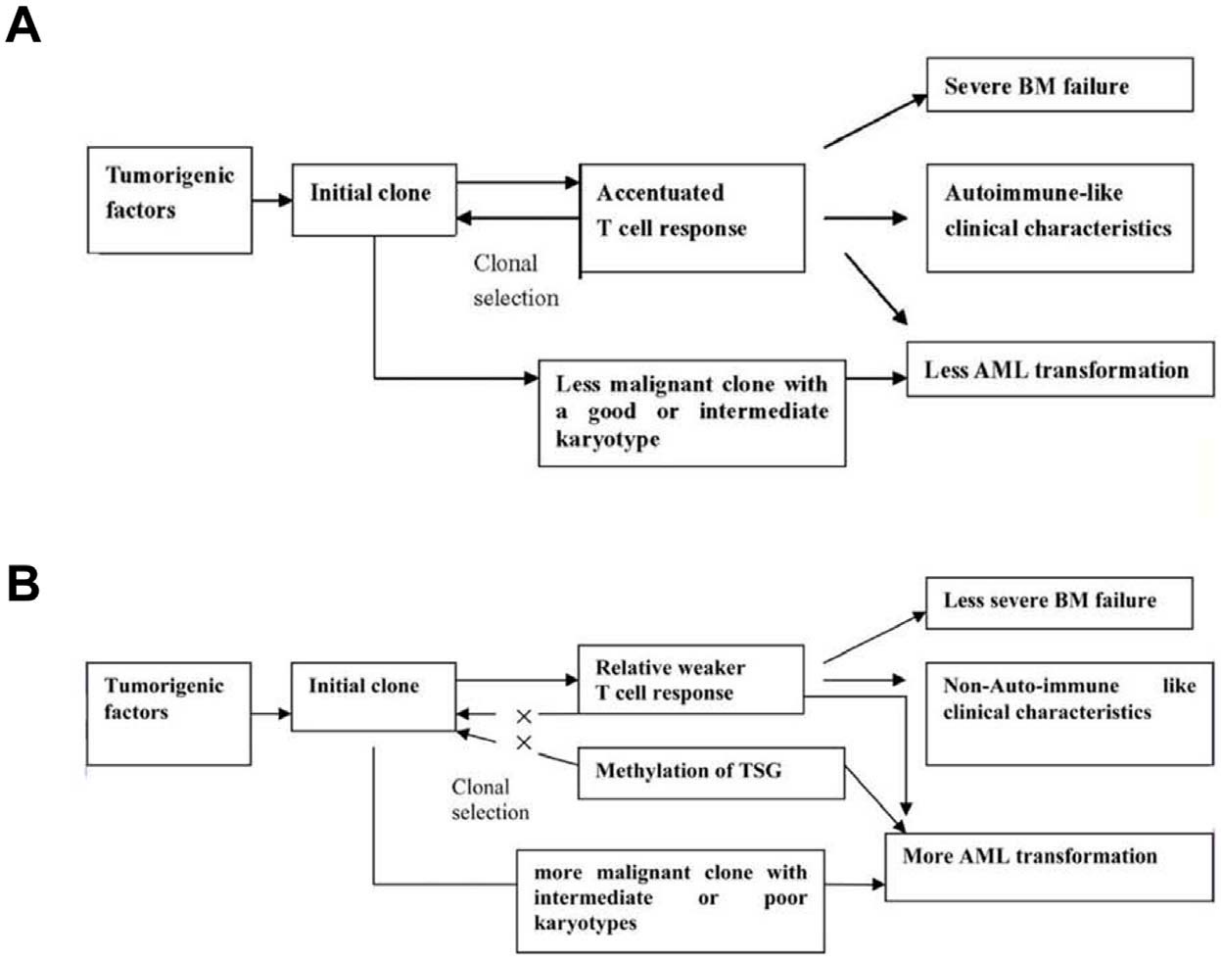
(A) Possible pathogenesis in younger patients; (B) Possible pathogenesis in older patients.

These characteristics suggested a trend towards bone marrow failure rather than disease progression for younger patients with MDS. What are the reasons for such distinct features in younger MDS patients? To put in another way, whether these characteristics imply some distinct age-associated pathogenesis in MDS is worth discussing We present the following hypothesis based on our observations (Figure 5). Both the female-to-male ratio and the prevalence of trisomy 8 were much higher in the younger patients than the older ones. It is well known that most autoimmune-related disorders are more commonly observed in females, with several reports indicating that MDS with trisomy 8 is often accompanied by autoimmune diseases, such as Behçet’s disease or adult Still’s disease [30], [31]. 16 patients with MDS were found to have both trisomy 8 and autoimmune diseases (unpublished data). In this study, the younger patients had frequent bone marrow failure and increased CFU growth after removal of the activated T cells *in vitro*, suggesting that younger MDS patients have a stronger self-immune surveillance reaction during the development and progression of MDS. The formation of initial MDS clones in the younger patients may induced a strong T-cell response, which in turn suppressed the MDS clones but simultaneously resulted in severe BM failure and an autoimmune-like clinical presentation. Lower incidence of abnormal chromosomes and better differentiation of CD34+ cells observed in the younger patients, may contribution to the existence of the initial MDS clones in conditions of strong T-cell attack. The stronger T-cell self-immune surveillance and performance of TSGs function (less p15^INK4B^ methylation) suppressed the development of clonal cells in younger patients, which yielded more low-risk patients, less AML transformation, and ultimately, significantly longer overall survival. On the contrary, the MDS malignant clones in the older patient might not be controlled by the weaker T-cell response (demonstrated by the presence of fewer CFUs compared with the younger patients after the deletion of the activated CD4 or CD8 cells) and the activated TSGs, although some of the tumor-targeted T cells (such as Tc1 and NK) number expanded. Predominantly malignant clones such as 5q−/−5 that were more frequently observed in older patients could be selected and expanded preferentially due to the weaker T-cell response in those patients. Weaker T-cell surveillance, poor differentiation of CD34+ cells and frequent activation of TSGs may result in more higher risk diseases, more rapid AML transformation, and ultimately, obviously shorter OS.

Matsuda et al [32] reported the difference in clinical features between Japanese and German patients with refractory anemia in MDS according to FAB classification. The results showed that Japanese patients were significantly younger, and have more severe cytopenias and lower risk of AML evolution, which are similar to our findings. However, compared to the studies reported by Kuendgen et al [5] and Matsuda et al [32], our study provides more useful information to shed light on the explanation of the difference between Western and Eastern countries based on the comparison between the younger MDS patients and the older ones. The differences between Western and Eastern MDS patients may be due to the different age distribution of the MDS patients. These distinctions would disappear if only comparing the older patients in China with all MDS patients in Western countries. CMML and 5q− syndromes have been less frequently diagnosed in Chinese patients than in Western patients. Chen has reported a CMML prevalence of 5.2% in 508 MDS cases [9], and the CMML subset also accounted for only 5.2% of all of the 502 MDS patients in our study. When the older patients were analyzed separately, however, the incidence of CMML reached 11.5% (22/191 cases), which approximates the rate from European (13.5%, 287/2124 cases) [33] and American (9.6%, 751/7827 cases) studies [1]. There have been few reports on the incidence of 5q-syndrome in China. Only one case with 5q-syndrome was diagnosed among the 351 MDS cases reported by Li (0.28%) [10], and only four cases with 5q-syndrome were diagnosed in our study with 502 MDS cases (0.75%). However, all four patients with 5q-syndrome were older than 60 years old, which implied that 5q-syndrome diagnosis accounted for 2.1% in the older group (4/191 cases), which is near the 2.2% rate (28/1243 cases) reported by Kuendgen et al [5]. Another important characteristic of Chinese MDS patients is that trisomy 8 and −5/5q− are the most and the least frequent chromosomal abnormality respectively. Trisomy 8 and −5/5q− abnormality accounted for 25.7% (35/136) and 12.5% (17/136) of the cases respectively in the study by Chen et al [9]. In Li’s report [10], similarly, trisomy 8 was also the most prevalent chromosomal abnormality (28.3%, 67/237 cases), and −5/5q− abnormality only accounted for 7.6% (18/237). In our study, trisomy 8 was also the most frequent abnormality (32.1%), and −5/5q− accounted for 16.3% of all of the cases, which is similar to other Asia data. When the data from only older patients were re-analyzed, however, the frequency of trisomy 8 declined to 21.6% and −5/5q− abnormities increased to 23.7%, similar to reports from Western countries [33].

In summary, MDS in China is diagnosed at an earlier age than in Western countries. Young patients present with less advanced disease, more slowly progress and significantly longer survival than older patients. We interpret these data as an indication that the self-immune surveillance mechanism may play a role in pathogenesis of younger patients with MDS.
